# Room temperature transpulmonary thermodilution (TPTD) with increased indicator 20 ml TPTD bolus compared with standard TPTD with 15 ml iced saline: a prospective observational study

**DOI:** 10.1186/cc14248

**Published:** 2015-03-16

**Authors:** W Huber, E Maendl, A Beitz, M Messer, T Lahmer, B Henschel, S Rasch, C Schnappauf, RM Schmid, ML Malbrain

**Affiliations:** 1 Klinikum rechts der Isar, Technical University of Munich, Germany; 2 Ziekenhuis Netwerk, Antwerp, Belgium

## Introduction

Use of ice-cold saline is assumed to provide best accuracy of TPTD to obtain the cardiac index (CI), global end-diastolic volume (GEDVI) and extravascular lung-water (EVLWI). However, room-temperature injectate might facilitate TPTD outside the ICU. A recent study [1] showed acceptable bias and percentage error (PE) for CI-room derived from TPTD with 15 ml room temperature saline compared with CI-cold using 15 ml iced saline for TPTD. However, GEDVI-room and EVLWI-room had borderline PE values close to 30%, and the bias of GEDVI-room markedly increased with higher values of GEDVI and in case of femoral CVC. Since imprecision of TPTD-room might be reduced by a larger volume of injectate, it was the aim of our study to compare CI, GEDVI and EVLWI derived from TPDT using 20 ml room temperature injectate with standard TPTD with 15 ml iced saline.

## Methods

In 31 patients 236 sets with two 20 ml TPTDs with 21°C and subsequently two standard TPTDs with 4°C saline were obtained using the PiCCO-2 device with the latest algorithm correcting GEDVI for femoral TPTD (Pulsion Medical Systems, Germany).

## Results

Fifteen female and 16 male patients, APACHE II score 21 ± 7. Mean values of CI (4.02 ± 0.98 vs. 3.96 ± 0.91 l/minute*m^2^; *P *< 0.001), GEDVI (800 ± 166 vs. 796 ± 163 ml/m^2^; *P *= 0.011) and EVLWI (10.3 ± 3.7 vs. 9.7 ± 3.6 ml/kg; *P *< 0.001) were slightly higher when measured at room temperature compared with cold saline. Mean bias and PE values were 0.06 ± 0.37 l/minute*m^2^ and 18.6% for CI, 4 ± 81 ml/m^2^ and 20.2% for GEDVI and 0.5 ± 1.1 ml/kg and 22.7% for EVLWI. Bias values in case of femoral compared with jugular indicator injection were not significantly different for CI (0.04 ± 0.41 vs. 0.11 ± 0.30 l/minute*m^2^; *P *= 0.161) and EVLWI (0.56 ± 1.19 vs. 0.42 ± 1.07 ml/kg; *P *= 0.492). Bias for GEDVI-room was significantly lower for femoral CVC compared with jugular indicator injection (-6.0 ± 81.1 vs. 18.9 ± 78.3 ml/m^2^; *P *= 0.008).

## Conclusion

Compared with previous data using 15 ml room-temperature injectate, our data with 20 ml room-temperature injectate in general provide acceptable bias and percentage error when compared with standard TPTD with 15 ml iced saline. This also applies for femoral CVC room-temperature TPTD which might also be related to a new PiCCO-2 algorithm correcting for femoral CVC site.
